# “Hey Siri! Perform a type 3 hysterectomy. Please watch out for the ureter!” What is autonomous surgery and what are the latest developments?

**DOI:** 10.4274/jtgga.galenos.2021.2020.0187

**Published:** 2021-02-24

**Authors:** İsmail Burak Gültekin, Emine Karabük, Mehmet Faruk Köse

**Affiliations:** 1 Department of Obstetrics and Gynecology, University of Health Sciences, Dr. Sami Ulus Training and Research Hospital, Ankara, Turkey; 2 Department of Obstetrics and Gynecology, Acıbadem University Faculty of Medicine, İstanbul, Turkey

**Keywords:** Autonomous surgery, robotic surgery, machine learning, skill learning, skill analysis

## Abstract

As a result of major advances in deep learning algorithms and computer processing power, there have been important developments in the fields of medicine and robotics. Although fully autonomous surgery systems where human impact will be minimized are still a long way off, systems with partial autonomy have gradually entered clinical use. In this review, articles on autonomous surgery classified and summarized, with the aim of informing the reader about questions such as “What is autonomic surgery?” and in which areas studies are progressing.

## Introduction

“Come on! Self-driving automobiles are okay, but autonomous surgery? Impossible!”

“Sooner or later, this dream will come true too, I'm sure ...”

“Oh God! Are we going to be unemployed?”

In the face of revolutionary technological developments, people react more or less like the above examples. Weaving workers in England at the dawn of the industrial revolution thought they would be unemployed after the invention of the moving shuttle, which is the key component of automatic looms. Subsequently they committed acts of violence in the form of breaking automatic looms and burning factories, known as the “Luddite Movement”. Despite these impulsive outbreaks, technological development continued and, as a result, a new balance was established between machine and human, and this inter-relationship has continued to develop to the present day. Looking from this perspective, we can think that weaving loom workers and future surgeons may not be too different.

When it comes to changing habits in the face of new products, I always think of our world-famous photographer, “Ara Güler”, who was once quite resistant to digital technology. The following exchange illustrates his attitude to the advances in photographic technology:

- fotograf.net: What do you use as a camera?

- Ara Güler: I can even take pictures with a Singer sewing machine.

- fotograf.net: Do you have a digital camera?

- Ara Güler: I do. I haven't even put my hands on it yet ...

However, years after this interview, according to his assistant, Fatih Aslan, one day he saw a digital camera in the hands of a photojournalist and looked at what he shot: “Awesome! Does this machine give such a result? I want the same right now, Fatih!” From then on until his death, he carried a digital camera with him, besides his famous Leica with a 35 mm Summicron lens.

All kinds of changes are painful processes, and no professional group is pleased with its waning importance or the idea of no longer being socially needed...human nature. However, we know historically that professions have changed, disappeared, new professions have emerged and a new equilibrium has been reached each time. Thanks to the autopilot and computer-aided flight assistance systems that have been used in aircraft for a long time and are now considered indispensable, airline transportation has become the most reliable form of travel today. Similarly, worker robots used in Amazon’s warehouses have distinct advantages such as high performance, 24-hour working and low error rates compared to human workers. For now, the best approach, taking into account the examples we have given above, is to look at the revolutionary developments in terms of “social benefit” rather than developing resistance. It is also important for interested parties - the surgeons - not to be left out of the process at the development stage.

Autonomous surgery has great similarities with autonomous driving, both in terms of development stages and definitions. Although autonomous surgery seems to be less popular and overshadowed by studies on autonomous driving, it is actually an area where investments and studies have been increasingly made in recent years. When we look at the companies and funds investing in this area, it is also surprising to see that the vast majority of them are the same companies and funds trying to develop autonomous driving. Apart from academic and state-sponsored studies, many international technology companies are also involved in autonomous surgery research. Highlights include Intuitive Surgical, Google, Verb Surgical (a joint venture of Google and Johnson & Johnson), Siemens, Toyota Research Institute, Autodesk, Honda, Intel, Comcast, Hewlett Packard, PhotoNeo, NVidia, National Science Foundation, and National Robotics Initiative.

For this review, a search was performed in PubMed and Institute of Electrical and Electronics Engineers databases with the keywords “surgery, robotics, surgical and medical robotics, skill learning, skill analysis, learning to perceive, machine learning, deep learning” and the articles were classified and summarized in order to give the reader information about questions such as what autonomic surgery actually is, what are the current developments and which areas are progressing.

## 1. Definitions and concepts

“Autonomous” means to be independent and capable of self decision-making. However, due to the ambiguity in the definitions and the lack of a standard, we encounter inappropriate use of the word in many cases. For example; the master-slave systems, which are now used in many surgical centers and are actually a high-tech motion repeater manipulator, have been mis-named as “robots”. This robot terminology, which is actually completely wrong, is unfortunately now in place. Therefore, it would be appropriate to create an autonomous terminology and classification to be used in surgery as soon as possible, in order to prevent a similar situation from occurring for autonomic surgery. The norms determining the degree of autonomy are not yet available for surgical systems, but the classification being used in the automotive industry can be helpful to give an idea. It is likely that future standards for autonomic surgery will be very similar.

The Society of Automotive Engineers standards are as follows:

Level 0: Full control by the human driver; just warnings provided by the machine.

Level 1: Hands are on the steering wheel with full control taken over immediately (hands-on).

Level 2: Hands are not on the steering wheel, but with full control taken over immediately (hands-off).

Level 3: Driving does not have to be monitored, driver may concentrate on anything else, but may take the control at any time (eyes-off).

Level 4: The driver can leave his seat and even sleep (mind-off).

Level 5: Fully autonomous driving, no human contribution required (driverless robotic taxis).

Only Level 0 to 2 systems (driver-assistance) are legally allowed in Europe today.

We do not know exactly how autonomous systems will achieve the maturity and reliability to enter clinical use and into which surgical systems autonomic features will be integrated (robotic laparoscopic devices, endoscopic robots, microbots, bio-inspired robots, etc). Classification according to the developmental order of the post-traditional surgical systems can give an idea about this issue (1):

### 1^st^ generation (Stereotaxic robots):

- PUMA 200 and Neuromate were developed for use in stereotaxic brain biopsies and tumor excisions as the first examples of this group. Subsequently, systems named SCARA robot, ROBODOC and AcroBot were developed and used for various stereotaxic procedures.

- These systems could not perform multiple consecutive procedural steps and needed the control of a human surgeon after each step. They were more suitable systems for use in tissues and areas with sharp boundaries (such as orthopedics) using mathematical/mechanical strategies. The definition of master/slave appeared during this period.

### 2^nd^ generation (Endoscopic robots):

- Mainly developed for use in soft tissue surgery and in difficult-to-reach surgical fields.

- The first sample, PROBOT (Imperial College, London) was developed to be used in the excision of pre-defined prostate tissue volumes.

In 1998 The Zeus (Computer Motion, Goleta, CA, USA) and in 2000 da Vinci (Intuitive Surgical Inc, Mountain View, CA, USA) emerged as two of the most famous robotic systems to date. The Zeus was used in Canada for the first beating-heart coronary surgery and in 2001 in the first trans-Atlantic telerobotic operation. In 2003, Intuitive Surgical bought The Zeus company, making da Vinci the only commercial product in this field. Within a relatively short period of time, many disciplines of surgery besides gynecology found a common ground and wide spectrum of applications with the da Vinci system. The limit/range of indications for gynecological operations performed with the da Vinci system also gradually expanded (2).

- Although da Vinci dominates the market as leader, a small number of other second generation robotic systems continue to be offered to the market. Among these, Raven (University of Washington) has made significant progress and has come to the fore as a programmable, modular system with a certain degree of autonomy, having surgical arms with 6-Degrees of Freedom (DOF). Another notable system, DLR MicroSurge (German Aerospace Center), has the advantage of using a common platform with Raven and sharing the same console, allowing surgeons to share teleoperative experiences.

### 3^rd^ generation (Bio-inspired robots):

- In the development process of minimally invasive surgery, single-port laparoscopy and natural orifice transluminal endoscopic surgery (NOTES) platforms have been developed claiming fewer intervention ports and less scarring.  The third generation surgical robots logged in, therefore, with snake-like systems having high articulation numbers to be used in NOTES.

- We can classify existing bio-inspired systems under three headings:

1. Flexible systems working with tendons:

- i-SNAKE (Imperial College, London), CardioArm.

2. Catheter navigation systems:

- Developed for percutaneous cardiovascular interventions. It is divided into two:

**- Mechanical:** Amigo (Catheter Robotics Inc.), Magellan (Hansen Medical Inc.).

**- Electromagnetic:** Niobe (Stereotaxis Inc.), CGCI (Magnetecs Inc.).

These are the robotic systems being guided by changing magnetic fields. Newer systems operating with regular magnetic resonance imaging (MRI) systems (keeping the investment costs at an appropriate level) are also under development.

3. Concentric tube devices:

- Since catheter robots have problems in transferring power to the end region as the length increases, concentric tube systems have been developed in order to overcome this problem. They are used especially in cardiovascular, neurovascular and respiratory system interventions, since they have very suitable structures for tubular organ groups. These systems are also suitable for fetal imaging and surgery during pregnancy due to the advantage of having very small footprint and needle size entry.

### 4^th^ generation (Microbots):

- These are systems that have been dreamed about since the science fiction movie “Fantastic Journey”, released in 1966.

- They comprise systems ranging from nanometer scale to a few millimeters in size.

- A popular example is the capsule endoscope (PillCam) system that moves passively through the digestive tract by peristaltic organ movement and wirelessly transmits image and data.

- New generation microbots will soon be put into clinical use. Studies continue on improvements in higher image quality, movement, location, telemetry, power, diagnosis and tissue manipulation. Biological systems are closely imitated to achieve these improvements. For example; insect, fish, snake, bacteria and parasite (flagella) movement patterns are being studied for locomotion. There are also highly promising studies on microbots in which locomotion is provided externally by the electromagnetic field of MRI systems (Metin Sitti et al.).

### 5^th^ generation (systems capable of autonomous decision making):

- Over time, we can expect that some of the surgical methods mentioned above in each of the technological generations will become more prominent, and some will decrease in popularity. Although current studies are mainly conducted on endoscopic robots, autonomous decision-making capability in the future will be able to be integrated into one or more systems that we cannot predict today.

The acceleration of autonomous surgery studies has been made possible by the development of deep learning algorithms. Artificial intelligence (AI) applications in medicine have often started in the form of enhancing human performance with computer systems, but are gradually evolving into systems that make more independent decisions. Successful examples include algorithms that reduce the error rates of pathologists in the diagnosis of cancer-positive lymph nodes from 3.4% to 0.5%, and help breast radiologists and breast surgeons to identify the high-risk group and reduce lumpectomy rates by 30%. While these applications are at the lower end of the spectrum, autonomous systems are located at the advanced stages of the spectrum of AI in medicine.

## 2. Current developments

When defining autonomic surgery, it is necessary to mention the complex relationship of elements including anatomy, surgical instruments and surgical techniques and also a plethora of subgroups within each element. If we use an analogy with autonomous driving so as to facilitate understanding; anatomy can be compared to the highway and all other environmental factors (buildings, trees, garbage bins, pedestrians, other vehicles, etc), the surgical instrument to the automobile, and surgical technique to the driving techniques and styles. In this analogy, the asymmetrical nature of both anatomy and highway components is immediately apparent. It is the anatomy (highway) component that causes the main difficulties in studies related to autonomous surgery and is responsible for the fact that the fully autonomous surgical systems will be delayed for a long time, even beyond our estimates. While surgical studies in real-time anatomical models are difficult enough, the in vivo situation is even more complex. Additional disadvantages include anatomical variations, respiratory movements, different tissue characteristics, similar textural properties of different organs, internal movements of tissue (positional changes caused by peristalsis), and deformation. For this reason, studies on autonomic surgery, which are still in the early stages of development, have focused and progressed almost entirely on surgical instruments, technical components and performing some predetermined limited subgroup tasks, such as suturing, knot tying, palpation and debridement.

The autonomous robotic systems, which are either at the project stage or under development are as follows (3):

1. The University of California Santa Cruz Surgical Robotics Platform (RAVEN);

- It is a system that uses stereo imaging.

- It has low degree motion planning and ability to detect grasper positions.

- It can perform multilateral debridement of phantom tissue.

2. Da Vinci Research Kit (DVRK);

- It can perform viscoelastic phantom tissue debridement.

- It can perform circular cutting in tissues with variable viscoelasticity.

- It has the ability to dissect in cryogel phantoms with ultrasound guidance.

3. RAVEN-II;

- It has a stereo tracking and near-infrared wavelength (NIR) imaging system.

- It recognizes pseudoneuroblastoma, creates a surgical plan and can perform semi-autonomous surgical ablation.

4. Smart Tissue Anastomosis Robot (STAR), John Hopkins University;

- It is the first system that has made significant progress in solving the problems of tissue movement, deformation and tissue similarity in soft tissue surgery.

- A 7-DOF arm is used, utilizes NIR imaging and has deep learning software for motion planning.

- Suture passing, knot tying and anastomosis features are quite improved. It has recently shown great success in semi-autonomous intestinal anastomosis. It can perform autonomic intestinal anastomosis in ex-vivo and in-vivo animal models, exceeding the performance of human surgeons.

- It can make straight cutting and tumor extraction in experimental animal tissue.

5. Bone drilling in ear surgeries that require precision such as cochlear implant placement.

6. Needle navigation in lung biopsies (Vanderbilt, University of North Carolina).

7. Brain tumor resection (Brain Tool Lab, Duke University);

- Using image data obtained from tomography, ultrasound and MRI systems, the robot arm precisely and accurately takes the appropriate position proper for the tumor tissue.

- The route of the surgical laser incision, control of the firing energy, safe firing and safe tissue ablation are provided using an autonomous algorithm in a closed-loop feedback.

None of the systems listed above are currently Food and Drug Administration (FDA) approved. For information purposes, the FDA can approve any medical device in two ways: Premarket approval (PMA) and 510 (k) permission. PMA covers the permits for completely new products, is costly (costs over $30 million per license) and requires a lengthy process for approval. The 510 (k) authorization includes approvals for improved versions of products that have already received PMA or similar products, is a much lower cost operation (costs less than $200,000) and can be completed in a relatively short time. FDA has not approved any medical device with the definition of a surgical robot to date. The da Vinci robotic system, which is commonly referred to as a robot with a misidentification, but is actually a master-slave system based on the principle of motion repetition, received FDA approval for the equivalent of “non-robotic laparoscopic equipment” in 2000 [via 510 (k)]. However, PMA approval will be required for autonomous surgery systems in the future. Recently an autonomous anesthesia device, named Sedasys by Johnson&Johnson, which can adjust the dose of sedation drug administered intravenously by monitoring parameters such as the patient's breathing, heart rhythm, and saturation, has passed FDA approval (with PMA) (4).

If we think of autonomic surgery as a big and complex problem, trying to solve it by dividing it into its small components is the right method to follow and studies continue in this way. In the near future, we will be able to find new generation robotic systems, which will facilitate the surgeon’s work during the operation, and which will have added some equipment with limited autonomy including the ability to perform certain tasks such as needle grasping at the right angle, suturing, knot tying, surgical ablation, and tumor resection with systems with varying degrees of autonomy.

We can summarize the main areas where the studies are concentrated as follows:

1. Identifying surgical instruments separately (segmentation) and real-time tracking of their movements and paths,

2. Grasping the surgical needle from the right angle and point,

3. Suturing,

4. Knot tying,

5. Biopsy needle guidance and precise bone drilling applications,

6. Real-time measurement of tissue flexibility and deformation, adaptive tissue cutting procedures,

7. Palpation,

8. Ablation, resection, debridement,

9. Evaluation of surgical skills and surgical training,

10. Computer-aided anatomy.

### 2.1. Identifying surgical instruments separately (segmentation) and real-time tracking of their movements and paths

One of the most critical issues for autonomic surgery is segmentation, which is the ability to identify the instruments in the surgical console separately, both from the surrounding tissues and from each other. In simpler terms, segmentation is the process of the computer program to understand which pixels belong to the living tissue, which pixels belong to the surgical instrument and the region of the instrument while evaluating the surgical area as a picture. This segmentation process is the most critical step in the realization of most autonomous tasks such as suturing, needle catching, knot tying, incision, ablation, and debridement. Real-time monitoring of segmentation and segmented structures are not easy processes due to reasons such as shadowing, specular reflections during surgery, fogging of the camera and lens, visual occlusion with blood and clots, and dynamic and complex changes of the surgical tissue area (5).

**Figure 1:**
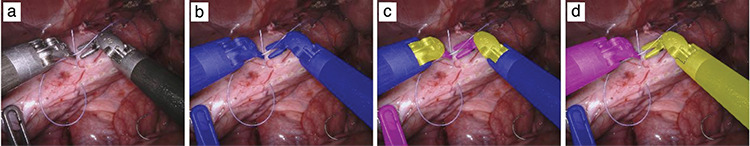
An image section taken during robotic surgery: a) Original image, b) binary segmentation (instruments blue, tissue red), c) multi-fold segmentation (three separate regions of the instrument can be identified: body, articulation joint, grasper tip), d) multiclass segmentation (each instrument can be defined separately) (5)

Successful segmentation studies have become possible as a result of developments in high-resolution, new generation cameras and deep learning algorithm architectures (U-Net, TernasusNet, LinkNet) (Figure 1).

The next step, after successful segmentation, is the ability to track the instruments in real-time. Real-time instrument tracking is getting faster and more precise, thanks to highly powerful computer processors and deep learning algorithms that successfully determine spatial location. The success rate can be increased further by linking the instruments to be used to machine learning systems and pre-educating them before the operation (6).

A secondary benefit and usage area of autonomous surgical instrument tracking is that the camera can take an autonomous position by tracking the instrument without the need for an assistant (cameraman robot). Some of the advantages of autonomous camera control compared to human assistants can be: cameraman robots do not get distracted, get tired, their stability does not decrease, they can follow better and more precise positions, and since there will be one less person in the operation team, it is ensured that the remaining team has more movement area in the operating room. In recent years, many cameraman robot systems have been introduced and put into use (EndoAssist, Aesop, EvoLap, etc). There are also systems that have human-machine interface differences (foot pedal, eye/head movements or voice control), whose common purpose is to take the task of holding the camera by relaxing the operator’s hand. However, these systems do not have instrument segmentation, motion tracking and autonomy capabilities. A laparoscopic system capable of autonomous monitoring has recently been commercially available (AutoLap, Medical Surgery Technologies, Yokneam, Israel). However, there is no published article about the sensitivity, success or deficiencies of this system yet (7).

### 2.2. Grasping the surgical needle from the right angle and point

Intelligent robotic surgical assistant systems, which are the next stage of master/slave systems and capable of performing some low-level surgical tasks autonomously, are on the horizon. The first of these surgical tasks is to grasp the needle autonomously. In this way, the problem of not grasping the surgical needle at the appropriate point/angle and the prolongation of the surgery time as a result of multiple regrasping attempts can be avoided. Three parameters are considered in surgical needle grasping: Manipulation, dexterity and torque metrics. Each of these parameters is important for a successful needle grasping (8).

Studies on robot gripping/grasping were first developed with the aim of facilitating the daily life activities of disabled people with motor deficits, long before being co-opted into autonomic surgery. While the first developed systems work with the help of comprehension databases, prepared with previously 3-D modeled objects (glass, fork, ball, etc), with the advancements in deep learning algorithms, successful grasping/capturing operations can be accomplished with machine learning today without the need for any preformed database (9).

**Figure 2:**
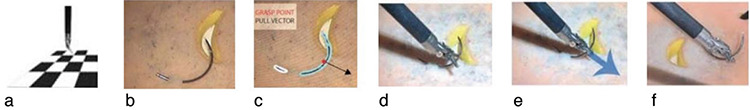
Steps in the process of grasping a surgical needle stuck into a tissue phantom at an appropriate angle and extracting it from the tissue by calculating the tensile force vector. a) Calibration, b) snapshot image creation, c) planning the segmentation and extraction phase, d) approaching phase, e) withdrawal phase, f) complete removal of the needle from the tissue (10)

**Figure 3:**
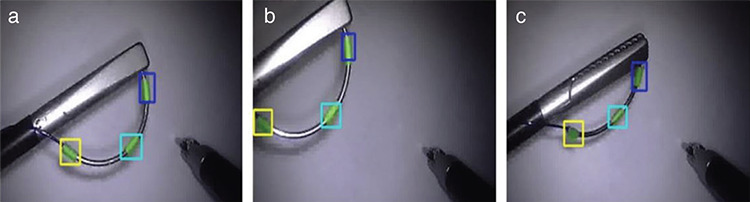
The segmentation regions (tail, body and tip) determined by the tracking algorithm that adapts to changing positions autonomously. a) Ideal position, b) position at the border of the endoscope’s visual field, c) the position at which the needle angle changes in the spatial plane. Segmentation of the needle was successful in all three cases (11)

Studies on grasping the surgical needle proceed on similar principles. The critical steps are the segmentation and movement planning of the tail, body and tip parts of the needle (10,11) (Figure 2, 3).

Various visual-based algorithms are used in robot capture studies. However, these algorithms are usually not suitable for surgery as autonomous robotic capture is largely unpredictable. In such cases, the surgeon’s choice among multiple capture points and different capture scenarios has emerged as an acceptable method. More successful systems can be developed after the data pool created by these choices in a cloud system is evaluated with deep learning (9).

### 2.3. Suturing

**Figure 4:**
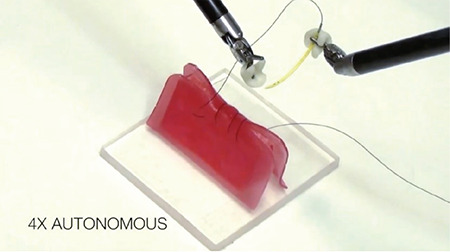
Fully autonomous suturing (needle grasping, needle transfer between instruments, suturing and knot tying) performed on a gel phantom after determining the starting point, distance between sutures and tissue depth (12)

One of the most time consuming procedures in robotic, minimally-invasive surgery is suturing, due to the lack of haptic (touch sensitive) feedback, limited range of motion, and narrow-angle field of view (12). After determining the basic elements of suturing (needle entry/exit points and depth of transition), autonomous needle capturing/grasping, suturing and knot tying will shorten the duration of the surgery, as well as causing less tiredness for the surgeon, less distraction and healthy planning of the next surgical stage. We will soon see some limited tasks added to the new generation of robotic surgical equipment, which we can call “semi-autonomous” or “restricted-authorized autonomy” (12) (Figure 4).

**Figure 5:**
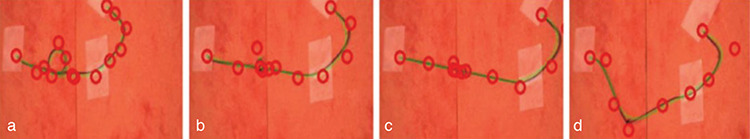
Identification of the suture thread along the entire length by segmentation and additionally determining the control points for motion tracking (tracking the dynamic relationship of the changing points in respect to each other is especially important during the knot tying phase). a) Multiple control points along the suture length, b) as a knot is tightened, the control points get closer and closer, c) after sufficient knot tightening, the knot with multiple points is redefined after a while, d) as a single control point (13)

There are three components required for a successful suturing process: needle, surgical thread and tissue. These three components should be defined separately by segmentation and their real-time movements should be followed in terms of both each individual component and the inter-relationship with the other two components. We have covered segmentation, and the grasping of the surgical needle in the previous section. Identifying the thread along its entire visible length in the surgical area and following it in real-time is essential for both suturing and the next step, knotting. Various methods have been proposed to identify and follow-up the suture material. Some studies have reported success with RGB plus imaging, which provides a high level of depth information, but RGB plus is not usually available in systems used today. In recent studies, stereo imaging, which is already widely used in existing systems and is starting to become standard, has been used and successful results have been obtained. Non-Uniform Rational B-Spline, a modeling algorithm for non-linear curves, can perform real-time suture thread identification and tracking by analyzing the stereo image pair used in existing systems (13) (Figure 5).

**Figure 6:**
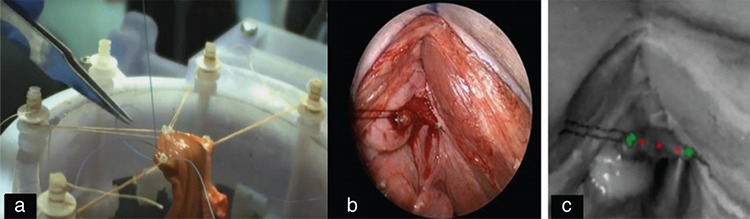
STAR procedures; a) intestinal anastomosis in ex-vivo pig tissue, b) in-vivo vaginal cuff (RGB camera view) after hysterectomy of pig, c) plenoptic camera view with the markings of the beginning, end and suture transition points (vaginal cuff closure was completed in about five minutes) (14) STAR: Smart Tissue Anastomosis Robot

The first example of successful suturing in living tissue is STAR, developed by John Hopkins University. It consists of a needle system activated by a modified Endo360 attached to a 7-DOF arm. Using NIRF and multispectral vision systems, it can perform the segmentation process in real-time. In its first results, published in 2014, it was reported that STAR performed intestinal anastomosis with acceptable accuracy (<0.5 mm) and deviation rates (0.2 mm). Subsequent studies continued on both 3-D models and live tissue, and successful preliminary results were published (14) (Figure 6).

### 2.4. Knotting

**Figure 7:**
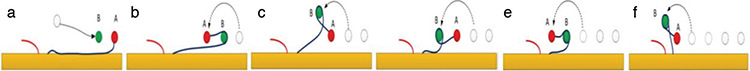
Trajectory of rolling arc looping (15)

Surgical knotting is a sequential procedure by rotating the suture thread held with instrument A around instrument B, then grasping the free suture tip with instrument B, and tightening the knot by pulling both instruments in opposite directions, as shown in Figure 7. This sequential task, which seems quite simple, is one of the reasons for the prolongation of the operation time in minimally invasive surgery. Difficulties during knotting are related to folding of the suture thread, narrow working area, limited depth perception, inability to feel the correct pulling force to be applied and insufficient experience. Studies on this subject can be divided roughly into two groups: new instrument designs that facilitate knotting and autonomous knotting algorithms using already existing instruments. The issue of new instrument designs, which are not widespread and far from developing a standard yet, will not be addressed here (15) (Figure 7).

Most of the studies were done with stereo cameras and two or three arms having at least 3-DOF, with standard grasper tips. Autonomous knot tying begins with the determination of the spatial planes of the needle entry and exit points and the step of removing the needle from the tissue and pulling the suture. Surgical arms, which are placed in opposite positions close to the needle entry and exit sites, are ready for the next step, ring formation. Often the distal end of the suture is left at a length of 25-35 mm. Two different methods can be used to wrap the suture on the opposite arm to form a ring: Spiral ring creation (slower) or rolling-arc method (faster). The knot is completed by grasping the distal end and pulling it mutually, after forming a suitable ring (15).

There are some studies on the use of skill transfer from humans which is a new learning method, mostly used in the field of rehabilitation robotics. Together with successful studies on this skill transfer with machine learning methods, which learns by analyzing the human surgeon’s movements in detail, a faster development of autonomic surgery may occur (16).

### 2.5. Biopsy needle guidance and precise bone drilling applications

Semi-autonomous biopsy needle guidance studies find use in three main areas: ablation, biopsy and brachytherapy. The common goal here can be summarized as minimizing tissue damage with increased sensitivity and smart maneuvers. Neuro-interventional procedures and spinal surgical procedures that require precise positioning and orientation, working with similar principles, are also clinical uses for these robotic systems. Clinical applications include ultrasound, MRI or luminous coherent tomography-guided interventions, cochlear implantation, motion compensation and orthopedic/neurological/radiosurgery robots. “MiniAture Robot for Surgical” procedures, Mazor Surgical Technologies, Israel, which provides instrument positioning and orientation in spinal surgery using a 6-DOF arm, SpineAssist robot used in spinal fusion surgery and precision percutaneous pedicle screwing, NeuroArm used under MRI guidance (University of Calgary, Canada) are examples of these systems (17,18,19,20,21).

### 2.6. Real-time measurement of tissue flexibility and deformation, adaptive tissue cutting procedures

**Figure 8:**
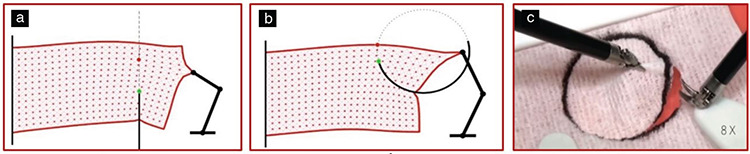
Autonomously calculated traction and counter-traction to overcome the problem of significant tissue deformation. a) Directing the biopsy needle tip towards the target, b) adjusting the needle exit point, c) completing the circular cutting process with high precision (21)

In surgery, almost all tissues except bone structures are deformable. Among the studies on deformable objects, studies of surgical cutting procedures in virtual environment have been especially prominent in recent years. Deformation calculations are used not only in cutting processes, but also in stereotaxic biopsies and suturing, in which the needle should pass through the correct line and exit from the correct point (Figure 8).

In order to solve the problem of tissue flexibility and deformation, we should consider three consecutive steps:

1. Modeling the textures with different material properties,

2. Analysis of traction and counter-traction responses,

3. Updating the new geometry and topology of the changed model after each cutting process.

In order for the tissue procedures to be suitable for real-time surgery, they must have 30 Hz refresh rates visually and 1,000 Hz as haptic feedback. In other words, the three steps mentioned above should be calculated over and over again each time and these calculations should be repeated at least 30 times per second in order to create a real-time perception. For this purpose, very high processing power is required. Studies often were done on mesh sheets, first in 2-dimensional models, then 3-dimensional models using multi-layered mesh. However, methods and hybrid models that do not use mesh have been recently studied. These are methods commonly referred to as point-based approaches. The precision and accuracy rates are significantly higher in point-based approaches than mesh models, especially in models with large deformations, but require proportionately higher processor power (22).

### 2.7. Palpation

Palpation is one of the main components of the physical examination. It is defined as the feeling and differentiation of parts with different degrees of hardness from the normal tissue, such as lymph nodes, nodules, and masses. Such lesions are the most common signs and symptoms encountered in the diagnosis of some diseases. Today, many robotic systems have force and position feedback features to different degrees and sensitivities. There are experimental systems used in the diagnosis of breast tumors, thyroid nodules and for determining the precise location of kidney stones during laparoscopy (17).

### 2.8. Ablation, resection, debridement

We mentioned in previous sections projects designed to perform surgical tasks such as ablation, resection and debridement with certain degrees of autonomy. RAVEN, which can perform multilateral debridement in phantom tissue, and DVRK, which can determine circular targets in viscoelastic tissue phantom, perform circular cutting and ultrasound-guided resection in cryogel phantom are the leading projects in this field. The drawbacks of these projects are that they have not been tested in animal tissues and they are limited to single-pass mechanical cutting. The problems of tissue motion, deformation and structural similarity in living tissues could not be solved in either of the projects. STAR is the first soft tissue surgery system that has found satisfactory answers to these problems. Using plenoptic and NIR imaging, the STAR platform can overcome the deformation problem by calculating metric co-ordinates in living tissue when marked with biocompatible markers. In a study using STAR and electrocautery, it was reported that electrical power, cutting speed and cutting depth were autonomously and precisely adjusted. Successful resection with STAR was also reported in the same study when the margin of resection of tumor tissue stored in pig living tissue was marked with biomarkers (21).

### 2.9. Evaluation of surgical skills and surgical training

“I climb the steepest mountains, jump over the high cliffs, dive deep into cold waters ...”

Everyone is a very good surgeon! - from the rookie assistant who has just completed his first month, to our seniors who are at retirement age. The lack of a system that objectively measures surgical skills is a large deficit here. The accepted standard today in the evaluation of surgical skills is peer review of either live surgery or video images during traditional surgery or retrospectively by a peer or more senior surgeon. However, there are question marks about the objectivity and accuracy/sensitivity issues that depend on human evaluators. The first studies to overcome this problem have been tried with surgical scenarios built on virtual reality systems. However, although it is indisputable that virtual surgery systems are very useful in education and gaining some skills, there are opinions that they cannot be assessed with the same criteria when it comes to surgical skill measurement.

**Figure 9:**
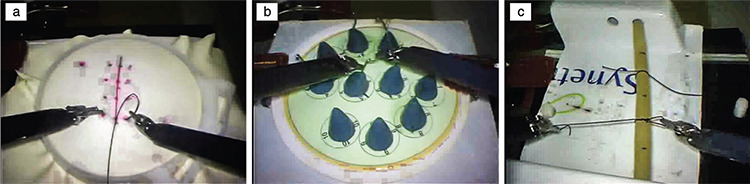
a) Suturing, b) needle passing, c) knotting tasks (parameters evaluated and reported by JIGSAWS) (22)

A new field of the objective measurement of surgical skills was developed during studies of deep learning in medicine. Developing autonomous skills and teaching/transferring surgical experiences to deep learning systems have shown promising results. Since it is possible to convert and analyze video recording and quantitative motion data into descriptive mathematical models in robotic surgery, these data can also be used for surgical training and extraction of automatic performance metrics (OPM). JIGSAWS (skill assessment set developed by John Hopkins University), a robotic surgery database open to everyone, is a system based on scoring of three parameters: Suturing, needle threading and knot tying. The JIGSAWS database was created by assessing these three parameters in a total of eight surgeons with different experience levels. Many parameters such as the spatial position (x, y, z), tracking of the surgical instrument tip, its rotation, linear velocities, angular velocities and the angle of the gripper tip are recorded as kinematic motion data in the JIGSAWS database (23) (Figure 9).

Studies in the field of measuring surgical skills can be divided into two main categories:

**1. Descriptive statistical analysis:** Skill analysis is performed using movement time, length of the path taken, motion pollution, curvature, semantic signs, instrument orientation, and vectoral force evaluations. However, very intensive manual engineering effort is required to properly arrange such optimal skill metrics. Another problem encountered here is the open-ended discussion about what is the best skill.

**2. Predictive model-based methods:** This is based on the principle of predicting skill measurement from the motion database. In itself it is divided into two parts; a) Descriptive and b) generative (machine learning). Recent studies have focused on the latter, the generative predictive model-based method. This model works on the basis of the analysis of OPM data (action data such as instrument and endoscopic camera motion tracking, energy modality usage times, etc) collected in live surgery with the da Vinci System’s recorder (dVLogger; Intuitive Surgical Inc) with machine learning algorithms. The data obtained here are extremely extensive and often contain details that can be missed by human observers.

One of the important differences between machine learning and traditional methods is the need for a reference for comparisons in traditional analysis. However, even among expert surgeons, there is often no consensus on what “good surgery” should be used as a reference and standard. There is no need for such a reference system in machine learning systems. Here, instead of a pre-built model, there are surgical templates shaped and learned with data itself. When the clinical outcomes of the patients are also added to the performance metrics in a machine-learning algorithm, such as intraoperative complications, postoperative complaints/findings, and length of hospital stay, a more objective evaluation can be obtained (24).

**Figure 10:**
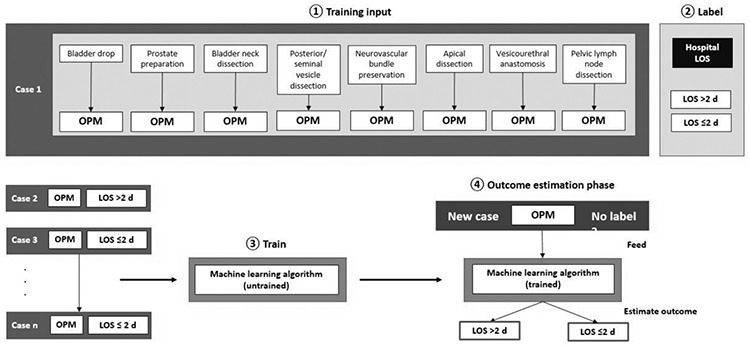
A machine learning model in which surgical performance in the surgical treatment of prostate cancer is measured with OPM and its effects on hospital stay are predicted (24) OPM: Automated performance metrics

If we examine a system based on the machine learning principle as an example, the subject might be a little clearer. In this example, the basic setup of a machine learning system for robotic radical prostatectomy operation is given. By using only the determined OPMs, the length of stay of the patient can be predicted with an accuracy rate of 87.2%. When some extra data concerning the patient are added (age, body mass index, prostate specific antigen level, prostate volume), the accuracy rate increased up to 88.5%. There is an ongoing study to determine the effectiveness of this model in predicting oncological and functional outcomes in the future and this will be possible after enough clinical data have been obtained over time. In this study, some different and unpredicted results were also obtained, which even experienced surgeons may not be aware of. For example; while the view that a balanced use of both hands is ideal for good surgery is widely accepted, this study found that master-level surgeons use their dominant hands more than novice surgeons (25) (Figure 10).

### 2.10. Computer-aided anatomy

Autonomic surgery will only be possible after the development of computer-assisted anatomy. Anatomy still remains a difficult problem to model digitally, not only because of the spatial volumes of highly complex physical structures but also because of their functional relationships with each other. Pioneering studies on this subject started with the studies of matching topographic anatomical information with radiological images by radiologists. Thanks to the developed algorithms, the location and segmentation of complex organs such as the pancreas, has recently become possible. There are studies in digital anatomy-related diagnoses and treatment assistance, radiotherapy planning, surgical simulation and estimation of tissue damage severity. In parallel with the widespread use of tomography and MRI, the number of such studies is also increasing (a study showing that studies involving multi-organ analyzes increased parallel to the number of tomography/MRI tests in the UK between 1995 and 2013).

The developments in this field will accelerate with the development and widespread use of 3-D modalities in medical imaging, the introduction of low artifact and high-resolution systems, and algorithms that make anatomically-consistent single organ and system segmentations.

This review will not go into details about the anatomical models, which is a large subject in its own right. Among these, 3-D MRI and probability calculations using deep learning algorithms and the generation of temporal and spatial data from 2-D MR and tomography sections stand out as promising methods, especially in multi-organ analyses (26). When anatomical studies are performed by dividing the body into regions, the most difficult region to analyze is the abdominal area. The reasons for this include the existence of more complex relationships between organs, peristaltic movements of the digestive system, respiratory movements, and changing anatomical relationships depending on factors such as age, disease and edema. Since deep-learning systems can process high-density data regarding abdominal anatomy more easily with increased processor speeds, there had been a significant increase in the number of publications in this area. When we look at the distribution of the studies by anatomical regions, the frequency of publications are as follows: the abdomen 24%, chest 8%, vertebrae 7%, pelvic organs (bladder, rectum, prostate, vagina) 7%, hand 3%, hip joint 3%, knee 2%, and elbow 1%. There are two freely accessible anatomical image databases (Tomography, MR, positron emission tomography) to be used in digital anatomy studies: National Institute of Health Cancer Imaging Archive and UK (United Kingdom) Biobank Imaging Study. It is an important advantage that these databases include normal anatomy as well as non-normal anatomy (cancerous organs, displacement of the remaining organs after surgery, etc) (18,27).

## 3. Ethical and legal issues

Although technical developments related to autonomic surgery are still in the very early stages, disproportionately intense ethical discussions are reported in the literature. Why is AI in surgery, which is still in its infancy, heavily discussed ethically and legally? We are not in a hurry, right? Actually, we are. Before the systems are put into use, ethical discussions should be completed and legal regulations should be started. While longer time is needed for the development of fully autonomous surgical systems, hybrid systems are on the horizon. A road map study was carried out in Geneva in 2006 by European Robotics Research Network and attention was drawn to these issues (28).

Currently, three areas where ethical debates are particularly heated are nuclear physics, genetic engineering and autonomous robotic systems. The first scientific symposium on robotics was held in 2004 and the World Robot Declaration, defining the basic features that a robotic system should have, was published. Accordingly, a robot;

1. should work on the basis of partnership with people,

2. should help people physically and psychosocially,

3. should contribute to the realization of a safe and peaceful society.

The vast majority of the publications regarding the ethical issues of AI and autonomous systems are related to medicine (e.g. surgical robots), civilian vehicles (e.g. autonomous cars) and military technologies (e.g. armed drones, targeting systems). Discussions and recommendations share similarities for all three areas.

The main discussion in the aforementioned systems is within the framework of “responsibility” (29). Responsibility has three elements:

**1. Accountability:** The ability to explain each transaction made. In the series of operations consisting of three parts, which are input, internal state (deep learning algorithm) and output, there is often no full transparency, especially concerning algorithms, for reasons such as the “black box” architecture specific to deep learning systems, and also non-disclosure of source codes because of trade secrets and copyright laws.

**2. Liability:** Who is primarily responsible for the system - manufacturer, operator or maintenance service provider?

**3. Culpability:** Who is guilty and should be punished in the event of a malfunction, such as an interruption of telecommunication during telesurgery? Again the candidates would be the manufacturer, operator or maintenance service provider.

As emphasized in Matthias’ (30) article and the European Parliament Resolution of 16 February 2017 on the rules of civil laws in robotics, there is great unpredictability in adaptive autonomous robots that may cause damage and the traditional laws of culpability would be unsuitable in such situations. One of the suggested solutions for liability is similar to that in autonomous cars, the surgeon sitting in the same room where the system operates, both as an observer and as a safety provider who can take full control any time. Another option would be to use a restricted system that only helps and assists the surgeon in routine operations, without giving full autonomy. Instead of “automatic machine learning” (uninterpretable, internal functioning, black box architecture), which is one of the discussion topics, “interactive machine learning” stands out as a more acceptable system in autonomous surgery. In the interactive machine learning system, the process of how the end-result is achieved can be monitored transparently, contributing also to the professional development of surgeons.

**4. Justification** of why a discovery is needed should be presented to the society for all inventions and developments with huge potential that will directly affect the society and will enter all our lives irreversibly. While the justification is fairly easy and understandable in some cases (such as laparoscopic vs conventional surgery), in some cases it can be really difficult (there is still an intense debate about the need and consequences of armed drones used in the military field). The situation concerning autonomic surgery is also more complicated than expected. Why should autonomic surgery be needed when there are human surgeons? While the debate continues about the need for autonomous systems for routine surgical practice, it is easier to justify why it is needed in some selected cases. Autonomous surgery can be truly life-saving in war zones, large infection outbreaks, space research stations and prolonged space flights. The intubation and resuscitation of seriously ill patients infected with “Severe acute respiratory syndrome” in 2002-2003 and during the ongoing Coronavirus disease-2019 pandemic are procedures that carry great risks for healthcare professionals. Robotic systems with deep learning algorithms that will facilitate teleoperative procedures (e.g. intubation) can be very useful in such cases.

Another issue is the problem of cybersecurity. In 2017 Bonaci et al. (31) published a case of hacking the control of a robotic system during a teleoperation by infiltrating the UK National Health System network. The issue was opened up to discussion and emphasized that security certificates should be redefined and strengthened, in much the same way as in military systems (31).

It is also useful to briefly mention a situation that is currently under discussion for telesurgery (and the same discussion will be held for autonomous systems in the future) called the Tort Law. If a lower performance operation is performed by a human surgeon while there is a possibility of performing a high performance operation with telesurgery or advanced autonomic surgery system by a more experienced surgeon, can the human surgeon be held responsible and blamed for this? It is highly controversial, but it will certainly raise the bar for surgical skills that surgeons must have.

The discussion on ethicolegal regulations and more should be completed before the development of a perfect autonomous surgical system that is completely safe and exceeds human performance.

## Conclusion

Autonomous surgery will happen sooner or later, although it may seem far away at present. First of all, we have to accept this. On the other hand, we need to do something in order not to adopt the position of the weaving workers during the Industrial Revolution. While there are dizzying speeds in technology, the relatively slow progress in autonomic surgery is actually an important chance for surgeons to adapt to and influence this process. Advances in AI and deep learning algorithms in non-surgical disciplines are quite advanced, especially in branches such as radiology, pathology, histology, embryology, and dermatology, which are predominantly image-based. AI systems that can evaluate mammography at the expert level, make serious progress in the diagnosis of diabetic retinopathy, and select the embryo with the highest chance of pregnancy in IVF cycles are rapidly coming into daily use. Most of our colleagues working in these disciplines either remained completely passive during the development phase of these systems or are still unaware of the advanced development stage the process has reached.

It would be an appropriate approach for surgeons to take active roles in the development process and trigger innovative initiatives, rather than passively waiting for the developed technology to be presented to them. The point that should be emphasized again and again is that the basic criterion underlying every development related to AI/deep learning and autonomic surgery in medicine is “social benefit”.

## References

[1] Ashrafian H, Clancy O, Grover V, Darzi A (2017). The evolution of robotic surgery: surgical and anaesthetic aspects. Br J Anaesth.

[2] Takmaz Ö, Gündoğan S, Özbaşlı E, Karabük E, Naki M, Köse F, et al (2019). Laparoscopic assisted robotic myomectomy of a huge myoma; Does robotic surgery change the borders in minimally invasive gynecology?. J Turk Ger Gynecol Assoc.

[3] Camarillo DB, Krummel TM, Salisbury JK Jr (2004). Robotic technology in surgery: past, present, and future. Am J Surg.

[4] Britton D. Autonomous surgical robots. Life Sciences Innovation: Law 321, 2016. Available from:.

[5] Shvets AA, Rakhlin A, Kalinin AA, Iglovikov VI (2018). Automatic Instrument Segmentation in Robot-Assisted Surgery using Deep Learning. 2018 17th IEEE International Conference on Machine Learning and Applications (ICMLA) Orlando, FL.

[6] Zhao Z, Chen Z, Voros S, Cheng X (2019). Real-time tracking of surgical instruments based on spatio-temporal context and deep learning. Comput Assist Surg (Abingdon).

[7] Khoiy KA, Mirbagheri A, Farahmand F (2016). Automatic tracking of laparoscopic instruments for autonomous control of a cameraman robot. Minim Invasive Ther Allied Technol.

[8] Liu T, Çavuşoğlu MC (2015). Optimal needle grasp selection for automatic execution of suturing tasks in robotic minimally invasive surgery. IEEE Int Conf Robot Autom.

[9] Jain S, Argall B (2016). Grasp detection for assistive robotic manipulation. IEEE Int Conf Robot Autom.

[10] Sundaresan P, Thananjeyan B, Chiu J, Fer D, Goldberg K (2019). Automated extraction of surgical needles from tissue phantoms. 2019 IEEE 15th International Conference on Automation Science and Engineering (CASE) Vancouver. BC, Canada.

[11] D’Ettorre C, Dwyer G, Du X, Chadebecq F, Vasconcelos F, de Momi E, et al (2018). Automated pick-up of suturing needles for robotic surgical assistance. IEEE Intl Conf Robot Autom.

[12] Sen S, Garg A, Gealy DV, McKinley S, Jen Y, Goldberg K. Automating Multiple-Throw Multilateral Surgical Suturing with a Mechanical Needle Guide and Sequential Convex Optimization. 2016 IEEE International Conference on Robotics and Automation (ICRA) 2016; DOI: 10.1109/ICRA.2016.7487611.

[13] Jackson RC, Yuan R, Chow DL, Newman W, Çavuşoğlu MC (2015). Automatic initialization and dynamic tracking of surgical suture threads. IEEE Int Conf Robot Autom.

[14] Leonard S, Wu KL, Kim Y, Krieger A, Kim PC (2014). Smart tissue anastomosis robot (STAR): a vision-guided robotics system for laparoscopic suturing. IEEE Trans Biomed Eng.

[15] Chow D, Jackson RC, Çavuşoğlu MC, Newman W (2014). A novel vision guided knot-tying method for autonomous robotic surgery. 2014 IEEE International Conference on Automation Science and Engineering (CASE) Taipei.

[16] Knoll A, Mayer H, Staub C, Bauernschmitt R (2012). Selective automation and skill transfer in medical robotics: a demonstration on surgical knot-tying. Int J Med Robot.

[17] Najarian S, Fallahnezhad M, Afshari E (2011). Advances in medical robotic systems with specific applications in surgery--a review. J Med Eng Technol.

[18] Moustris GP, Hiridis SC, Deliparaschos KM, Konstantinidis KM (2011). Evolution of autonomous and semi-autonomous robotic surgical systems: a review of the literature. Int J Med Robot.

[19] Majewicz A, Wedlick TR, Reed KB, Okamura AM (2010). Evaluation of robotic needle steering in ex vivo tissue. IEEE Int Conf Robot Autom.

[20] Moreira P, Patil S, Alterovitz R, Misra S (2014). Needle steering in biological tissue using ultrasound-based online curvature estimation. IEEE Int Conf Robot Autom.

[21] Opfermann JD, Leonard S, Decker RS, Uebele NA, Bayne CE, Joshi AS, et al (2017). Semi-autonomous electrosurgery for tumor resection using a multi-degree of freedom electrosurgical tool and visual servoing. Rep U S.

[22] Wang M, Ma Y (2018). A review of virtual cutting methods and technology in deformable objects. Int J Med Robot.

[23] Wang Z, Majewicz Fey A (2018). Deep learning with convolutional neural network for objective skill evaluation in robot-assisted surgery. Int J Comput Assist Radiol Surg.

[24] Zia A, Essa I (2018). Automated surgical skill assessment in RMIS training. Int J Comput Assist Radiol Surg.

[25] Hung AJ, Chen J, Gill IS (2018). Automated performance metrics and machine learning algorithms to measure surgeon performance and anticipate clinical outcomes in robotic surgery. JAMA Surg.

[26] Cerrolaza JJ, Picazo ML, Humbert L, Sato Y, Rueckert D, Ballester MÁG, et al (2019). Computational anatomy for multi-organ analysis in medical imaging: A review. Med Image Anal.

[27] Lin B, Sun Y, Qian X, Goldgof D, Gitlin R, You Y (2016). Video-based 3D reconstruction, laparoscope localization and deformation recovery for abdominal minimally invasive surgery: a survey. Int J Med Robot.

[28] Veruggio G. EURON Roboethics Roadmap. EURON Roboethics Atelier, Genoa, Feb 20-March 3, 2006. Available from: (July 1, 2006)..

[29] O'Sullivan S, Nevejans N, Allen C, Blyth A, Leonard S, Pagallo U, et al (2019). Legal, regulatory, and ethical frameworks for development of standards in artificial intelligence (AI) and autonomous robotic surgery. Int J Med Robot.

[30] Matthias A (2004). The responsibility gap: ascribing responsibility for the actions of learning automata. Ethics Inf Technol.

[31] Bonaci T, Yan J, Herron J, Chizeck HJ (2017.). Experimental analysis of cyber security attacks on teleoperated surgical robotics. In preparation to the ACM Transaction on Cyber-Physical Systems.

